# Environmental Control of Adult Neurogenesis: From Hippocampal Homeostasis to Behavior and Disease

**DOI:** 10.1155/2014/808643

**Published:** 2014-12-18

**Authors:** Sjoukje D. Kuipers, Clive R. Bramham, Heather A. Cameron, Carlos P. Fitzsimons, Aniko Korosi, Paul J. Lucassen

**Affiliations:** ^1^Department of Biomedicine, University of Bergen, 5009 Bergen, Norway; ^2^Department of Biology, University of Bergen, 5020 Bergen, Norway; ^3^K. G. Jebsen Centre for Research on Neuropsychiatric Disorders, 5009 Bergen, Norway; ^4^National Institute of Mental Health, National Institutes of Health, Bethesda, MD, USA; ^5^Swammerdam Institute for Life Sciences (SILS), Center for Neuroscience, University of Amsterdam, Science Parc 904, 1098 XH Amsterdam, The Netherlands

There are few fields in neuroscience that have witnessed a faster development than the field of adult neurogenesis in the past decade. The discovery of stem cells present in the adult brain that give rise to new neurons has raised a lot of interest as it changed current concepts of brain plasticity and possible strategies for brain repair. While neurogenesis, today, has become a well-acknowledged phenomenon, many open questions remain. In this special issue, we have compiled a selection of articles that address several timely topics related to neurogenesis and discuss some of the unresolved questions concerning the functional relevance of adult neurogenesis, its regulation, and its role in the diseased brain.

The history of the field of adult neurogenesis is filled with controversies. By the end of the nineteenth century, largely due to influential scientists like y Cajal [1], it was firmly believed that no new neurons were added to the adult mammalian brain. A central dogma in neuroscience was that brains of mammals remained structurally constant from soon after birth. Neurogenesis was believed to occur only in early development and to rapidly decrease shortly thereafter. In the early 1960s, ground-breaking studies challenged this well-accepted doctrine by reporting the presence of newborn cells in various brain structures of young and adult rats, including the cerebral cortex, hippocampus, and olfactory bulb [2, 3]. These reports, however, were essentially ignored by the scientific community, and it was not until the end of the twentieth century, more than 100 years after the initial formulation of y Cajal's tenacious dogma, that a novel concept could develop. In the late 1990s, a series of papers initiated an explosion of research on the existence, function, and implications of adult mammalian neurogenesis. Over the years, accumulating evidence has since established adult neurogenesis as a concept, and it is now widely accepted that the adult brain is far from being fixed but is rather a highly plastic organ in which new neurons are indeed added to the existing network throughout life in all mammals including humans. An overview of the controversial history of adult neurogenesis is reviewed in this issue by E. Fuchs and G. Flügge.

Today, we know that neurogenesis occurs in the adult central nervous system throughout life in at least a few discrete regions, like the hippocampus and subventricular zone. From rodents to primates, neurons are continuously produced in the subgranular zone of the hippocampal dentate gyrus. New neurons are also generated in the subventricular zone, the largest germinal zone of the adult mammalian brain, from which they extensively migrate along the rostral migratory stream into the olfactory bulb.

A highly dynamic process, adult neurogenesis is further regulated by several endogenous as well as exogenous factors, such as age, exercise, (early) stress, and disease [4–7]. Environmental stimuli (e.g., diet and stress) and social interactions can greatly affect adult neurogenesis at multiple levels. These include proliferation, fate specification, migration, integration, and survival. In this issue, T. Murphy and colleagues address dietary interventions as effective environmental modifiers of brain plasticity. The authors evaluate the gap in our mechanistic understanding and discuss recent findings from animal and human studies reporting beneficial effects of dietary factors on cognition, mood and anxiety, aging, and Alzheimer's disease. Finally, they discuss the obstacles involved in harnessing these promising effects of diet on brain plasticity as seen in animal studies, into effective recommendations for humans and interventions to promote brain health. P. Peretto et al. review how the social environment impacts adult olfactory bulb neurogenesis. They discuss how social behaviors related to reproduction promote the proliferation and integration of newborn neurons into functional circuits. These social influences on adult olfactory bulb neurogenesis may ultimately enhance individuals' fitness, as these “fresh” neurons contribute to critical activities such as parental behavior and partner recognition. Environmental influences on neurogenesis may already occur before conception but also continue during the peripartum period (pregnancy, birth, and lactation) which is characterized by numerous alterations in maternal neuroplasticity and neurogenesis, crucial for the physiological and mental health of the mother.

K. M. Hillerer et al. review common peripartum adaptations in mothers' physiology and behavior, focusing on changes in neurogenesis and their possible underlying molecular mechanisms. From conception onwards, our physical and social environments trigger a series of physiological responses that modify our later responsivity by acting on the genetic blueprint to adjust developmental and lifelong programming of mental function. Early life represents a particularly sensitive period to the programming influences of environmental factors. Interestingly, the immune system plays an important role in the communication between the human body and its environment. While this holds true during both early development and adulthood, preliminary evidence suggests that early-life activation of the immune system can affect hippocampal neurogenesis and increase the risk for psychiatric disorder development later on. K. Musaelyan et al. further examine the effects associated with such immune system activation during early life, providing evidence to support a neurogenic hypothesis of immune developmental programming.

One of the most important and extensively studied environmental influences on neurogenesis is stress, both acute and chronic. Whereas brief stressful challenges appear beneficial for brain plasticity, allow adaptation, and in some instances even increase neurogenesis, chronic stress exerts deleterious inhibitory effects on plasticity, especially in the hippocampus. These detrimental influences are largely attributed to the elevation of glucocorticoids, through molecular mechanisms that are still not entirely clear. In the final part of their review, E. Fuchs and G. Flügge provide an overview of the influences of stress and stress hormones on the regulation of adult hippocampal neuroplasticity. The deleterious actions of chronic stress on neurogenesis have led to speculations regarding involvement of hippocampal neurogenesis in the aetiology of depression as well as antidepressants' mode of action. In this issue, P. Rotheneichner et al. analyze the relationship between the various mechanisms of action of electroconvulsive therapy (ECT), a powerful second-line treatment for major depression disorders that strongly stimulates neurogenesis. They explore the intricate interactions between electroconvulsive shocks, hypothalamic-pituitary adrenal axis, neurogenesis, angiogenesis, and microglia activation as well the role of neurogenesis in age-related changes of ECT response in mice. J. L. Pawluski et al. instead explore the effects of fluoxetine, the most common antidepressant in the treatment of mood disorders, on hippocampal neuroplasticity and neurogenesis in female rats. They provide new evidence indicating that different modes of administration (oral versus minipump) of this antidepressant differentially modulate hippocampal neurogenesis in adult female rats.

Although somewhat counterintuitive, neurogenesis is especially responsive to neurodegeneration affecting the hippocampus. In fact, emerging evidence suggests that impaired neurogenesis may represent an early event in the course of various neurodegenerative disorders. From a functional perspective, adult neurogenesis provides new cells which are important for structural plasticity and network function. Newborn neurons in the adult hippocampus and subventricular zone participate in memory processing, mood regulation, and olfaction, functions commonly impaired in subjects suffering from Parkinson's (PD) or Alzheimer's disease (AD), two of the most common neurodegenerative disorders in humans. Disturbed regulation of new neuron production may exacerbate network vulnerability and promote early subtle disease manifestations. In this issue, M. Regensburg et al. summarize and interpret existing data on adult neurogenesis in patients with Parkinson's disease and related animal models. A fundamental process in PD and AD, neuroinflammation, has been implicated in the progression of both diseases. Microglial cells, the major orchestrator of the brain inflammatory response, promote neuroprotective or neurotoxic microenvironments, thus controlling neuroprogenitor cell proliferation and neuronal fate. K. J. Doorn et al. address whether early microglial activation may play a role in the development of hippocampal pathology in Parkinson's disease and study the proliferative responses occurring in the hippocampus of PD patients. Remarkably, they use double-labeling techniques to show that the proliferation in the PD hippocampus is largely due to microglial cells. A. Sierra et al. explore the interplay between microglia and neurogenesis and discuss both the beneficial and detrimental roles of microglial cells on adult hippocampal neurogenesis regulation, in the context of stress, aging and neurodegeneration, and particularly Alzheimer's disease. Finally, M. W. Marlatt et al. discuss cell proliferation observed in the hippocampus of AD patients and describe the close proximity of dividing cells to amyloid plaques. Using novel triple immunocytochemical protocols, they further demonstrate that it is not astrocytes but rather the microglia cells, which appear to underlie the proliferative response in the AD hippocampus.

This special issue includes 11 exciting articles covering various aspects of adult neurogenesis, from its physiological regulation to its relevance for the pathophysiology of various brain disorders. We are convinced that this selection of papers will help the readers gain a better understanding of the crucial role of adult neurogenesis in both the healthy and diseased brain.

*Sjoukje D. Kuipers* *Sjoukje D. Kuipers* *Clive R. Bramham* *Clive R. Bramham* *Heather A. Cameron* *Heather A. Cameron* *Carlos P. Fitzsimons* *Carlos P. Fitzsimons* *Aniko Korosi* *Aniko Korosi* *Paul J. Lucassen* *Paul J. Lucassen*
